# Intravaginal electrical stimulation for the treatment of pelvic floor dysfunction: a systematic review and meta-analysis

**DOI:** 10.3389/fneur.2024.1378494

**Published:** 2024-08-13

**Authors:** Rongrong Chen, Rui Wang, Yanmei Yu, Kun Zhao, Juebao Li

**Affiliations:** ^1^Center for Rehabilitation Medicine, Rehabilitation and Sports Medicine Research Institute of Zhejiang Province, Department of Rehabilitation Medicine, Zhejiang Provincial People's Hospital, Affiliated People's Hospital, Hangzhou Medical College, Hangzhou, Zhejiang, China; ^2^School of Basic Medical Sciences, Zhejiang University School of Medicine, Hangzhou, China

**Keywords:** intravaginal electrical stimulation, pelvic floor disorders, meta-analysis, systematic review, PRISMA

## Abstract

**Background:**

Intravaginal electrical stimulation (IVES) has been explored as a potential treatment for pelvic floor disorders (PFDs), although its efficacy remains a subject of debate. We aim to conducted a comprehensive meta-analysis of relevant trials.

**Methods:**

This meta-analysis was performed under the PRISMA 2020 guideline. We meticulously searched for randomized controlled trial (RCT) studies in various databases, including PubMed, Cochrane Library, EMBASE, and ClinicalTrials.gov, spanning from inception to March 6, 2023. All studies included one treatment group of intravaginal electrical stimulation and the diseases spectrum of the studies involved different kinds of PFDs, including urinary incontinence, overactive bladder, etc. Risk of bias charts were used to assess the risk of bias in the studies and forest plots were used the demonstrate the overall effects.

**Results:**

Our analysis encompassed a total of 13 RCT studies. In most of the assessed PFD cure outcomes, the results demonstrated positive effects of IVES therapy, as indicated by the following findings: daily voiding frequency (MD = −1.57, 95% CI = −3.08 to −0.06, *I*^2^ = 68%,), nocturia (MD = −1.07, 95% CI = −2.01 to −0.13, *I*^2^ = 71%), Pad test, and Urinary incontinence. Nevertheless, the data concerning the impact of IVES therapy on the quality of life of individuals with PFDs did not confirm these positive results.

**Discussion:**

In light of the insufficiency in both the quality and quantity of the included studies, it is premature to draw a definitive conclusion regarding the efficacy of IVES therapy for treating PFDs. Nonetheless, our study does provide several pieces of evidence in support of the potential therapeutic effects of electrical stimulation therapy in this context. We recommend that further research in this area be conducted to provide more conclusive insights into the efficacy of IVES therapy for PFDs.

**Systematic review registration:**

https://www.crd.york.ac.uk/prospero/, identifier: CRD42023442171.

## 1 Introduction

Pelvic floor disorder (PFD), also known as pelvic floor dysfunction, is characterized by a range of symptoms associated with the malfunction of the pelvic floor, including urinary incontinence (UI), pelvic organ prolapse (POP), and sexual dysfunction, etc. (1). Brief introduction of these subtypes are as follows.

Among UIs, there're SUI, UUI, and MUI (2). Stress urinary incontinence (SUI) characterizes the involuntary release of urine prompted by heightened abdominal pressure. Considering different treatment options for SUI, surgical interventions have evolved over time. In contrast, urge urinary incontinence (UUI) manifests as involuntary urine leakage accompanied by a strong sensation of urgency and an immediate need to urinate that cannot be delayed. Mixed urinary incontinence (MUI) encompasses a combination of SUI and UUI symptoms.

As for POP, it is a prevalent condition among women (3). Pelvic organ prolapse refers to the descent of one or more pelvic organs from their anatomical position, resulting in the formation of a bulge within the vaginal region, known as a prolapse. Normally, the pelvic organs are supported by the muscles and connective tissues of the pelvic floor, which ensure their proper placement and functioning. Its occurrence has been on the rise in tandem with the overall increase in life expectancy. A range of treatment options, both conservative and surgical in nature, are available to address this condition.

It is estimated that women have a 1 in 4 face a lifetime risk of experiencing PFD, while in many cultures these conditions may have a connection with stigmatization and women tend to suffer with the symptoms silently (4). However, previous researches (4) have pointed out that treatment of these symptoms can have positive effects on their quality of life and sexual satisfaction.

The risk factors of PFDs involved a large range of etiologic factors. Usual factors included increasing age, weight, parity, and a history of hysterectomy (5). Previous studies had proved that as women age the prevalence increases, with >40% of women older than 40 years old experiencing urinary incontinence (6). Therefore, it is recommended that annual screening for PFDs in women should be promoted regardless of whether risk factors were present.

According to previous guidelines (7), the spectrum of treatments for pelvic floor disorders spans from lifestyle and behavioral therapy to surgical interventions, pharmaceuticals, and the use of medical devices (8–10). Among the conservative treatment modalities, pelvic floor muscle training (PFMT) has been recommended as the first-line approach to manage PFD symptoms, particularly stress urinary incontinence (SUI) (11). However, the evidences for electrical stimulation (ES) of the pelvic floor muscles (PFMs) were variable and couldn't reach solid conclusions (11). Therefore, further comparison is needed to be done in this subject.

Pelvic floor muscle training (PFMT) is a structured exercise program which improves pelvic floor muscle strength, endurance, power, relaxation, or a combination of these. It has been reported that PFMT can prevent the symptoms of PFDs and is now considered to be applied before using other intravaginal devices (11). Meanwhile, previous study (12) had pointed out that PFMT combined with the additional treatment using PFMT devices, its effects can be maximized and improved.

Previous studies have proposed three theorized mechanisms for PFMT (13). The first and dominant mechanism is that PFMT exercises the levator ani muscle to increase the cross-sectional area of the key support muscle underlying the urethra (14). The PFMT programs based on this mechanism are called “Kegel's exercises” or pelvic floor muscle (PFM) exercises. The second mechanism targets the urethral striated muscle to maximize the awareness of the timing of the PFM (15) and the corresponding programs are called “the Knack,” “stress strategy,” and “perineal lock.” The third mechanism aims at the transversus abdominis (TrA) muscle to strengthen the core muscle, and the PFMT programs reliant on this theorized mechanism are typically referred to as core muscle training (16). However, it is unclear how much evidence is available to support these theorized mechanisms and further studies are needed to clarify this subject.

Intravaginal electrical stimulation (IVES) is a method of passive muscle activation through the direct stimulation of weakened muscles or nerve fibers. The mechanism of action of electrical stimulation is complicated. It consists of a direct action inducing pelvic floor striated muscle hypertrophy and activation of the detrusor inhibitory reflex arc.

Though IVES is recommended by some physicians (11), its actual efficacy remains controversial. Data from systematic review (17) indicated that there was insufficient evidence both in favor of and against the use of the IVES therapy for women with SUI, probably due to the variability in the interventions of the included trials and the inadequacy of trial data (18). As for surgical interventions, they were not recommended unless other treatments have proven ineffective (11).

Trials concerning IVES as therapies for women has proliferated in recent years, and as for provide guidance for clinical practice, there is a need for more comprehensive assessments of IVES, including a thorough exploration of side effects, comparisons with other therapeutic approaches, and evaluations of IVES as a monotherapy or in combination with other treatments. Moreover, heterogeneities are needed to be solved and explained between these trials. Hence, our study aimed to investigate on whether IVES can treat female PFD syndrome. Furthermore, we aim to assess the efficacy of intravaginal ES, whether administered in conjunction with other treatments or as a standalone therapy, in comparison to no intervention, sham ES, or any other conservative treatment.

## 2 Methods

This systematic review and meta-analysis followed the Preferred Reporting Items for Systematic reviews and Meta-Analyses (PRISMA) 2020 guidelines (19). We've registered this program in Prospero before the study (the registration ID was CRD42023442171). Meta-analysis is a secondary data analysis method that relies on existing academic literature, and it does not involve direct experimentation with human subjects. As such, no new ethical review or approval was required for this research.

### 2.1 Data sources and search strategy

We searched PubMed, Cochrane library, EMBASE, ClinicalTrials.gov from inception to March 6, 2023. Search terms included those related to electric stimulation, intravaginal, and their variants. The full search strategy is provided in Supplementary Table 1. We also extracted relevant articles that met the inclusion criteria for randomized controlled trials included in previous systematic reviews or meta-analyses.

### 2.2 Eligibility criteria

#### 2.2.1 Types of studies

Only randomized controlled trials (RCTs) available as full-text articles were considered for inclusion.

#### 2.2.2 Types of participants

Female patients diagnosed with pelvic floor dysfunction, including urinary incontinence, overactive bladder, inability to voluntarily contract the PFMs efficiently. However, due to the limitation of quantity of the studies involving both PFDs and IVES, the spectrum of PFDs only involves urinary incontinence (UI), detrusor instability, overactive bladder (OAB). Some subtypes of PFDs, such as pelvic organ prolapse (POP) cannot be fully explored due to insufficient data.

#### 2.2.3 Types of interventions

The following comparisons were made: IVES as a monotherapy or in combination with other treatments vs. no active treatment, sham ES or other conservative treatments for pelvic floor dysfunction.

#### 2.2.4 Types of outcomes

The primary outcomes were objective cure outcomes, defined as cure outcomes that were measured with objective measures and less susceptible to a variety of factors (including results of pad test, daily voiding frequency, PFM strength, etc.), and subjective cure outcomes, defined as cure outcomes that were measured with subjective measures like scales and more susceptible to a variety of factors, including results of International Consultation Incontinence Questionnaire-UI Short Form (ICIQ-SF, ICIQ-UI-SF), urgency. The secondary outcomes were outcomes concerning life quality, including results of incontinence quality of life scale (I-QoL), Spinal Cord Injury Quality-of-Life Measurement System (SCI-QoL), etc.

### 2.3 Data extraction

A 2-step data extraction process was conducted. During the first stage, according to the study titles and abstracts, two independent reviewers with no interests made decisions in a standardized data extraction form based on the eligibility criteria. During the second stage, studies that passed the previous stage were downloaded, and the full texts were reviewed. Any discrepancies or disagreements were resolved through discussion and consensus. The following information was extracted from each included study: first author, year of publication, country, population characteristic (age, number parity), intervention (type, frequency, pulse width, intensity, and duration), comparison, and outcomes.

### 2.4 Risk of bias assessment

Studies meeting the inclusion criteria were evaluated for methodological quality to assess the risk of bias employing the Cochrane Collaboration's risk of bias tool as described in the Cochrane Handbook for Systematic Reviews of Interventions; each quality item was graded into three levels: low risk, high risk, or unclear risk (20). The quality assessment covered the following domains: allocation concealment, bias in the allocation process, bias in the results (integrity and authenticity), bias in the measurement process, and selective outcome reporting. Independent assessment by two reviewers was performed in the study and disagreements in the process were solved by a third reviewer.

### 2.5 Data synthesis and analysis

Continuous outcomes were used for statistical efficacy analysis using Hedge's standardized mean difference (SMD) for pad test, PFM strength, quality of life and urinary incontinence episodes and mean difference (MD) for frequency, maximal cystometric capacity, nocturia, and urgency with 95% confidence intervals (CI) with the random effects model for pooling estimates for each analysis. Subgroup analysis between IVES monotherapy and IVES in combination with other traditional treatments were performed. Binary outcomes were analyzed using rate ratio (RR) with 95% CI with random effects model either, but no analysis for specific outcomes were included in the final article due to limited quantity of studies included. The significance of the pooled effects was evaluated by a *Z*-test, and a *P*-value of < 0.05 was considered significant. *I*^2^ statistic was used to examine overall heterogeneity between studies and values higher than 50% were defined to have high heterogeneity. All statistical analyses were performed using Review Manager 5.3 (Nordic Cochrane Center) and Stata software version 15.1 (StataCorp, USA).

## 3 Results

### 3.1 Study selection and characteristics of included studies

**Figure 1:**
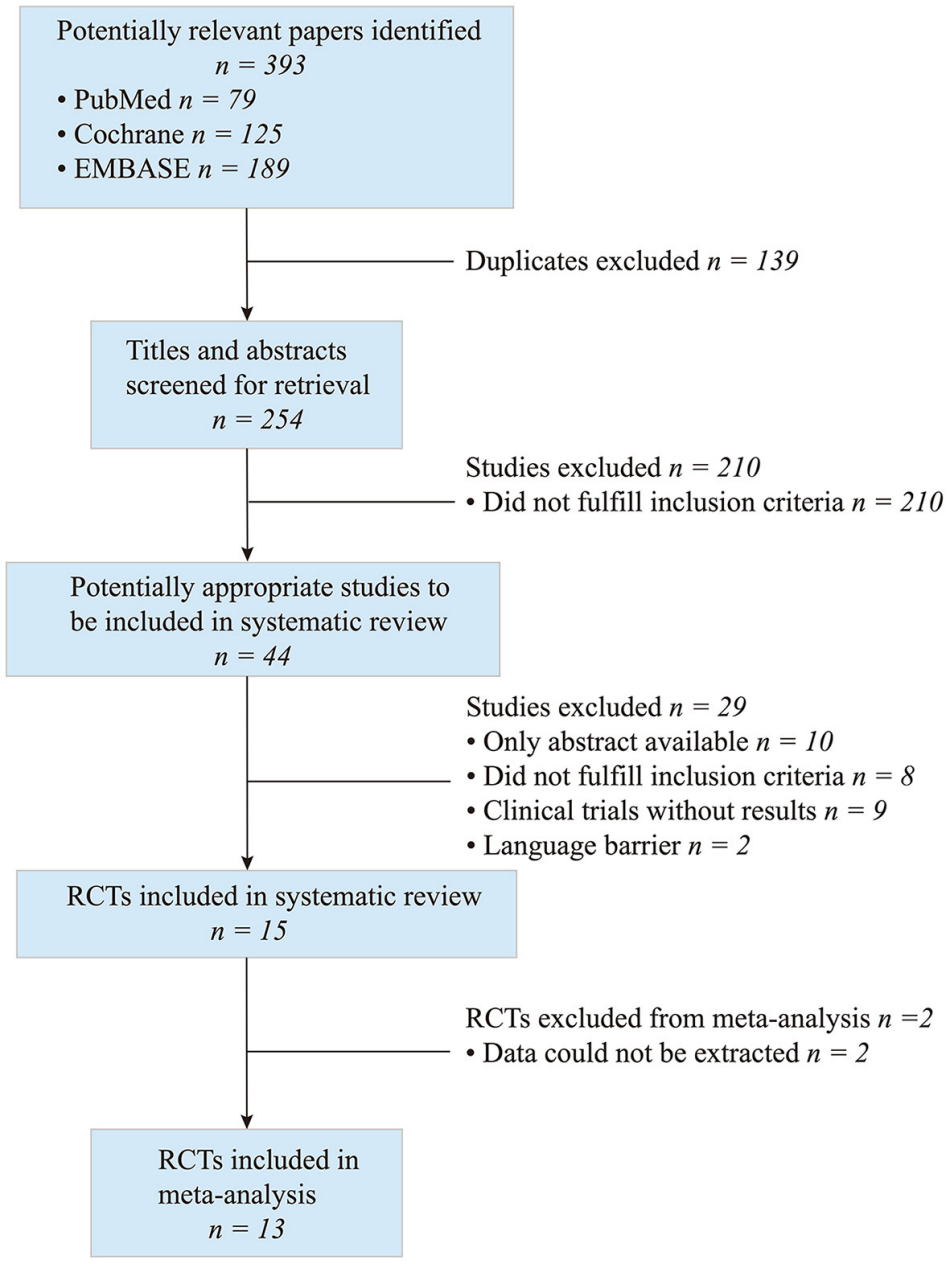
Screening process of included studies.

A total of 393 articles were identified by the electronic search. The titles, abstracts and full texts revealed that 13 met the inclusion criteria (Figure 1) and included 559 participants with pelvic floor muscle dysfunction.

The characteristics of included studies are presented in Table 1. Most of the studies included are single-centered and the studies come from the following countries: six in Brazil, two in Turkey, one in China, one in USA, one in Netherland, one in Denmark, and one in Poland. Target diseases were urinary incontinence (UI) (21–27), including stress urinary incontinence (SUI), mixed urinary incontinence (MUI), urge urinary incontinence (UUI), overactive bladder (OAB) (28–30) and other PFDs (31–33). Studies on nonspecific diseases concerning lower urinary tract were also included (23, 31, 33). As for intervention of the studies, nine studies involved IVES monotherapy (21, 22, 24–26, 28, 29, 32, 33) and the rest four studies involved other treatments in combination with IVES treatment, including surface EMG (sEMG) (27, 31), pelvic floor muscle training (PFMT) (23, 31), and bladder training (BT) (30).

**Table 1:** The characteristic of included studies.

**References**	**Country**	**Diagnosis**	**Experiment group**					**Control group**			**Outcome measures**
			**Number**	**Parity**	**Age**	**Intervention**	**Stimulation parameters (pulse width, frequency, duration)**	**Number**	**Mean Age (years, SD or range)**	**Control**	
Smith (25)	USA	SUI	9	1.7 (0–3)	53 (26–72)	IVES	Device: Unknown electrical stimulation parameters Stimulation parameters: frequency: 50 and 12.5 Hz; work-rest cycle: 5–10 s; pulse width: 300 μs; intensity: increased each month to a maximum of 80 mA Duration: 15, 30, 45, and 60 min (increasing), twice a day, 4 months	9	48 (36–70)	PFMT	PAD test
Spruijt et al. (26)	Netherlands	SUI (12.5%) UUI (16.7%) MUI (70.8%), postmenopausal age	24	2 (0–6)	72 (65–92)	IVES	Device: Urogyn 8900 ES system Stimulation parameters: frequency: 50 Hz (SUI) or 20 Hz (UUI); work-rest cycle: 1–2 s; pulse width: 2 s; intensity: gradually increasing up to the level of tolerable discomfort (0–100 mA) Duration: 30 min, thrice a week, 8 weeks	11	74 (65–86)	PFMT	PAD test PFM strength
Amaro et al. (21)	Brasil	MUI	20	/	49 (41–79)	IVES	Device: Dualpex Uro 996 Stimulation parameters: frequency: 4 Hz; work-rest cycle: 2–4 s; pulse width: 0.1 μs; intensity: according to patient discomfort level feedback Duration: 20 min, thrice a week, 7 weeks	20	47 (40–78)	Sham IVES	PFM strength
Wang et al. (29)	China	OAB	24	Unclear	Unclear	IVES	Device: Periform Stimulation parameters: frequency: 10 Hz; work-rest cycle: 10–5 s; pulse width: 400 μs; intensity: varying with patient tolerance (minimum 20 to 63 mA and maximum 40 to 72 mA) Duration: 20 min, twice a week, 12 weeks	21	Unclear	Placebo	Pad test Voiding diary
Ozdedeli et al. (28)	*Turkey*	OAB	16	3.0 (0–5)	57.5 (36–78)	IVES	Device: Myomed 134 and Enraf-Nonius Stimulation parameters: frequency: 5 Hz; work-rest cycle: unclear; pulse width: 100 μs; intensity: maximal level tolerable Duration: 20 min, thrice a week, 6 weeks	15	60.0 (37–78)	Trospium hydrochloride	Voiding diary
Terlikowski et al. (27)	Poland	SUI	64	Unclear	46.9 ± 6.8	TVES+sEMG	Device: Dualpex Quark^®^ Stimulation parameters: frequency: 10–40 Hz; work-rest cycle: 15–30 s; pulse width: 200–250 μs; intensity: maximal level tolerable Duration: 20 min, twice a day, 8 weeks	29	45.6 ± 7.9	Placebo+sEMG	Quality of life Pad test Voiding diary
Correia et al. (22)	Brazil	SUI	15	2.80 ± 0.94	59.86 ± 4.82	IVESG	Device: Dualpex Quark^®^ Stimulation parameters: frequency: 50 Hz; work-rest cycle: 4–8 s; pulse width: 700 ms; intensity: maximal level tolerable Duration: 20 min, twice a week, 12 weeks	15	60.13 ± 9.35	No intervention	PAD test PFM strength Quality of life
Lúcio et al. (31)	Brazil	Lower urinary tract symptoms	10	1.5 (0–3)	42 (27–54)	PFMT+ EMG-BF+intravaginal NMES	Device: Dualpex Quark^®^ Stimulation parameters: frequency: 10 Hz; work-rest cycle: 60 s; pulse width: 50 ms; intensity: at the participant's maximum tolerated intensity Duration: 30 min, twice a week, 12 weeks	10	43.5 (25–51)	PFMT+ EMG-BF+sham sacral NMES.	Pad test Voiding diary PFM strength ICIQ-SF
Elmelund et al. (23)	Denmark	UI	14	2 (1–2)	59 (49–67)	PFMT+IVES	Device: CefarPeristim Pro^®^ Stimulation parameters: frequency: 40 Hz (intermittent) and 10 Hz (continuous); work-rest cycle: 5–10 s; pulse width: 250 μs; intensity: at the women's maximum tolerated intensity Duration: 30 min, once a day, 12 weeks	13	47 (36–56)	PFMT	ICIQ-UI-SF Voiding diary Pad test Quality of life
Mateus-Vasconcelos et al. (32)	Brazil	PFD	33	3.2 (2.3)	55.6 (10.3)	IVES	Device: Dualpex Quark^®^ Stimulation parameters: frequency: 50 Hz; work-rest cycle: 5–10 s; pulse width: 200 ms; intensity: according to the patient's discomfort level feedback Duration: 20 min, once a week, 8 weeks	33	53.5 (14.0)	PFMT	ICIQ-SF
Rodrigues et al. (33)	Brazil	Inability to voluntarily contract the PFMs efficiently	17	3.00 [2.40–4.17]	57.43 ± 9.761	IVES	Device: Dualpex Quark^®^ Stimulation parameters: frequency: 50 Hz; work-rest cycle:8–16 s; pulse width: 300 ms; intensity: according to the patient's discomfort level feedback Duration: 20 min, once a week, 6 weeks	18	58.57 ± 13.15	IVVS	PFM strength
Yildiz et al. (30)	*Turkey*	OAB	29	No: 1 (3); 1–3:20 (69.0); ≥4: 8 (27.6)	55.24 ± 10.57	BT+IVES	Device: Enraf Nonius Myomed 632 Stimulation parameters: frequency: 10 Hz; work-rest cycle: 5–10 s; pulse width: 100 ms; intensity: 1–100 mA (according to the patient's discomfort level feedback) Duration: 20 min, three days a week, 8 weeks	29	56.44 ± 11.62	Bladder Training	Pad test PFM strength Bladder diary Quality of life
Ignácio Antônio et al. (24)	Brazil	UI	28	2.8 (2.1)	53 (12)	IVES	Device: Dualpex Quark^®^ Stimulation parameters: frequency: 50 Hz; work-rest cycle:5–10 s; pulse width: 200 ms; intensity: according to the patient's discomfort level feedback) Duration: 20 min, once a week, 8 weeks	33	54 (13)	No intervention	ICIQ-UI-SF

Studies included were of high quality. There were low risk in randomization method, data integrity and selective reporting of all 13 studies (21–33), allocation concealment of six studies (21, 23, 24, 26, 30, 33), blind method for participants of 12 studies (21–23, 25–33), and blind method for outcome measurer of 10 studies (21–24, 27, 28, 30–33).

### 3.2 Results of individual studies and syntheses

#### 3.2.1 Objective cure outcomes

**Figure 2:**
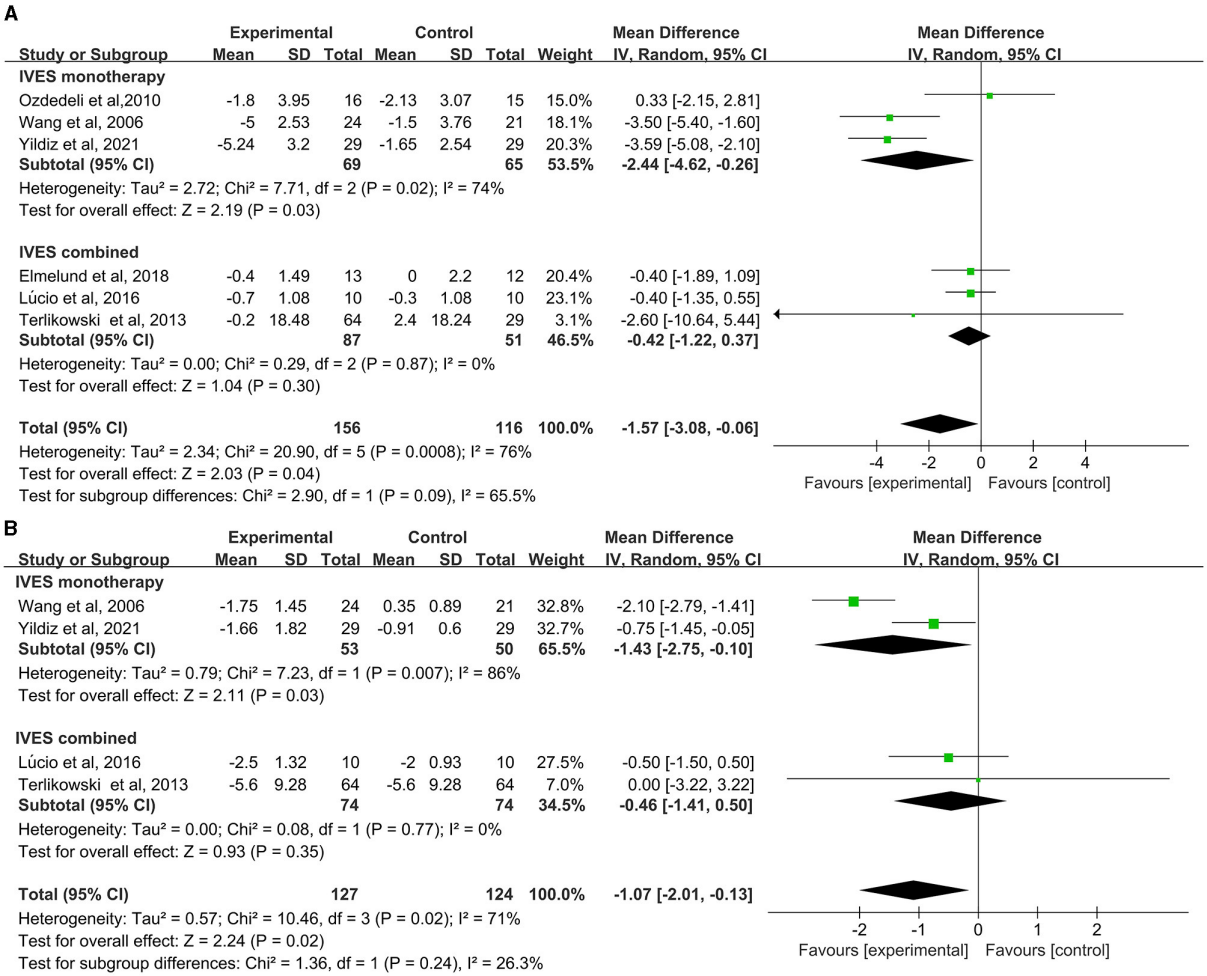
Forest plot of daily voiding frequency or nocturia outcome between the ES and other conservative treatment groups. **(A)** Daily voiding frequency; **(B)** nocturia.

Data from six studies suggested that intravaginal ES had a more preferable effect in improving daily voiding frequency than no active treatment or sham ES (MD = −1.57, 95% CI = −3.08 to −0.06, Figure 2A), but significant heterogeneity was found (*I*^2^ = 96%, *P* = 0.0008). Subgroup analysis of these studies regarding the comparison between IVES as a monotherapy and IVES in combination with other therapies (e.g., PFMT, EMG), the results confirmed that groups treated with IVES monotherapy showed significant improvements compared to groups combined with other therapies (Figure 2A).

Data from four studies showed that intravaginal ES is significantly effective in reducing nocturia than placebo or sham ES (MD = −1.07, 95% CI = −2.01 to −0.13, Figure 2B). Heterogeneity was also found in this analysis (*I*^2^ = 71%, *P* = 0.02). Subgroup analysis of the comparison between IVES as a monotherapy and IVES in combination with other therapies confirmed that IVES monotherapy contributed to a significant reduction in nocturia (Figure 2B), yet heterogeneity was still found in the comparison (*I*^2^ = 86%, *P* = 0.03).

**Figure 3:**
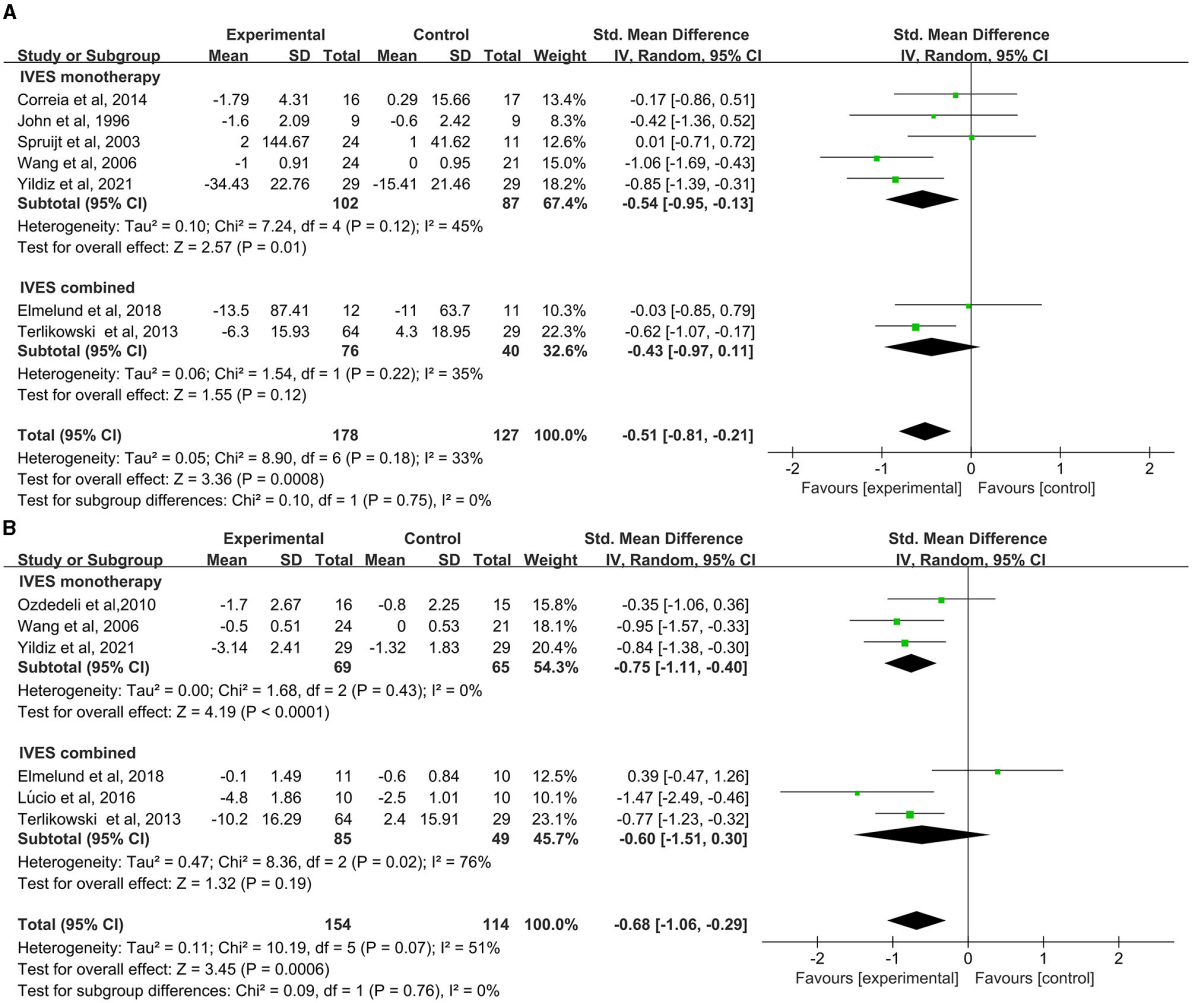
Forest plot of pad test or urinary incontinence result between the ES and other conservative treatment groups; **(A)** pad test. **(B)** Urinary incontinence.

Results of the pad test concerning seven studies was analyzed. Data suggested that IVES contributed to significant improvement in pad test results (MD = −0.51, 95% CI = −0.81 to −0.21, Figure 3A) as well as IVES monotherapy (MD = −0.54, 95% CI = −0.95 to −0.13) while no significant effect was found in IVES in combination with other therapies (MD = −0.43, 95% CI = −0.97 to 0.11) and no heterogeneity was found.

Status of urinary incontinence based on patients' voiding diary (including frequency of urine loss, incontinence episodes) of six studies suggested significant improvement in urinary incontinence (Figure 3B), with minor heterogeneity found in the comparison (*I*^2^ = 51%, *p* = 0.07). Subgroup analysis found out that IVES as a monotherapy can contribute to improvement in urinary incontinence (Figure 3B) without heterogeneity found in this comparison.

Data from three studies indicated that intravaginal ES is not significantly effective in improving maximal cystometric capacity than sham ES or trospium hydrochloride (Table 2). Heterogeneity was also found (*I*^2^ = 71%, *p* = 0.03). Subgroup analysis was not applied since only three studies was analyzed.

**Table 2:** The overall table of the rest of the results.

**Outcomes**	**Subgroups**	**Heterogeneity**		**Subgroup effect (MD, 95% CI)**
		*I* ^2^	***p*** **-value**	
ICIQ-UI-SF	-	0%	0.66	−0.58 [−2.95, 1.79]
Maximal cystometric capacity	IVES monotherapy	-	-	130.50 [49.42, 211.58]
	IVES combined	69%	0.07	46.07 [−77.53, 169.67]
	Total	71%	0.03	75.68 [−20.45, 171.81]
PFM strength	IVES monotherapy	92%	< 0.00001	−0.18 [−1.33, 0.96]
	IVES combined	-	-	0.82 [−0.10, 1.74]
	Total	91%	< 0.00001	−0.01 [−1.00, 0.97]
Quality of life	IVES monotherapy	0%	0.42	−0.69 [−1.02, −0.36]
	IVES combined	84%	0.01	−1.10 [−2.24, 0.04]
	Total	69%	0.01	−0.88 [−1.36, −0.40]
Urgency	-	96%	< 0.00001	−3.32 [−7.37, 0.73]

In this table, the results of Maximal cystometric capacity and PFM strength belonged to the objective cure outcomes and the results of ICIQ-UI-SF and urgency belonged to the subjective cure outcomes, while quality of life is sorted into the secondary outcome of this meta-analysis. All data was analyzed under random effect model and subgroup analysis was conducted if heterogeneity was found in this group.

PFM strength (including objective and subjective PFM strength) of six studies was also compare between IVES group and control group. Data showed no significant improvement for IVES group (Table 2) with high heterogeneity (*I*^2^ = 91%, *p* < 0.00001). Meanwhile, subgroup analysis of IVES as monotherapy showed similar results and no significant difference was found.

#### 3.2.2 Subjective cure outcomes

Data from two studies showed no significant improvement in scores of urinary incontinence scales (including ICIQ-UI-SF, ICIQ-SF) and no heterogeneity was found (Table 2). Self-reported urgency of three studies using subjective measurements (including VAS scale, 4-day voiding diary, etc.) indicated no significant difference between both groups (Table 2). No further subgroup analyses were performed as there was not enough studies in these outcomes.

#### 3.2.3 Secondary outcome

Data from five studies suggested that IVES could lead to significant reduction in the impact of PFDs on quality of life (Table 2), while heterogeneity was detected (*I*^2^ = 69%, *p* = 0.01). Subgroup analysis showed that IVES monotherapy contributed to lesser PFDs impact on QoL level in women than sham ES or no active treatment with no heterogeneity, while IVES combined with other treatments (including PFMT, EMG) didn't show significant effect.

## 4 Discussion

The major goal of this meta-analysis is to analyze published trials concerning the effects of intravaginal electrical stimulation on women with Pelvic floor disorders, and determine whether IVES is an effective method to treat these clinical conditions. Pooled data from included trials have given several instructive conclusions as follows. The 11 studies involved in the analysis were of high quality.

Regarding most objective outcomes measuring the treatment of PFD symptoms (including daily voiding frequency, nocturia, pad test results, and UI status), pooled data from published clinical trials confirmed the positive effects of IVES therapy. As shown in subgroup analyses, it was confirmed that IVES monotherapy contributed to a more preferable effect than IVES combined with other treatments. However, intravaginal ES failed to yield statistically significant improvement in maximal cystometric capacity, PFM strength between the ES and other conservative treatment group. The small quantity of studies included may explain the negative result.

Regarding the two subjective outcomes included in this study, all kinds of analyses failed to yield any significant difference, whether for or against. The reason behind might be the insufficiency of the quantity and quality of evidence included as well as the lack of large-sample multi-centered random clinical trials. Additionally, the subjectivity of the measuring process of these outcomes may explain part of the results.

Concerning these outcomes, high-quality clinical trials had given similar results in support of ES therapy. Previous randomized controlled trials (34–36) have found significant improvement from baseline concerning objective outcome leakage episodes, pad testing, vaginal muscle strength, etc. Meanwhile, results of subjective outcomes including visual analog scores of urinary incontinence, King's Health Questionnaire score (34) also showed significant improvement from baseline, which made a supplement to our results.

Though presenting many evidence in support of intravaginal ES as a treatment for pelvic floor disorders, this meta-analysis didn't reach the conclusion that intravaginal ES therapy is better than other therapies, such as PFMT or drug intervention. Apart from the limited quantity of studies included that involved different kinds of other therapies, the failure of subgroup analysis of IVES combined with other therapies to show significant results also posed a question to whether IVES reigned over other therapies in treatment of PFM dysfunction. Opposite evidences were found in previous systematic reviews. Data from meta-analysis (37) suggested that it was too early to say whether ES is similar or superior to other active treatments like PFMT in effectiveness. Another explanation for this problem may stem from the fact that our search strategy is too broad. Pelvic floor dysfunction is a group of urinary dysfunctions caused by different pathophysiological mechanisms, and most of the participants in previous studies on this topic have been stress incontinence/mixed urinary incontinence. We believe that heterogeneity in the included study populations may be one of the reasons for the reduction in the significance of the results. Moreover, it is suggested (27, 37, 38) that intravaginal electrical stimulation may result in device-related adverse effect (including urinary tract infection, vaginal infection, etc.) and there was insufficient data to determine whose adverse effect tended to be larger. However, the studies analyzed in this systematic review included trials concerning ES with non-implanted devices other than IVES, which may affect the results. Nevertheless, the reasons of low significance of our results are worth exploring and future researches of these hypotheses mentioned above are needed.

As for the secondary outcome, we found improvement in quality of life (QoL) in both IVES and IVES monotherapy, which established a strong connection between quality of life in UI (or other PFM dysfunctions) women and IVES therapy for PFM dysfunction treatment.

Regarding this result, previous studies had given similar results. In the RCT conducted by Kargar Jahromi et al. (35), researchers observed a significant improvement from baseline in PFMT group for incontinence quality of life at 8.5 weeks, pointing out the connection between the alleviation of PFD symptoms like UI and quality of life. Meanwhile, data from Cavkaytar et al. (39) indicated a similar elevation in QoL level from baseline using different scales (including QoL form, Oxford scale, PGI-I) in group of home-based Kegel exercises. However, data from meta-analysis on IVES or ES therapy were rare in this subject.

The limitations of the present study should also be acknowledged. Firstly, the substantial level of heterogeneity suggests that the results obtained should be interpreted with caution. Secondly, due to the limitation in the relevant evidence, only a small number of studies were identified. Thirdly, the population of women with PFM dysfunction was not further subdivided into UI and OAB, etc. and other treatments combined with IVES (including PFMT, IVVS, EMG, etc.) were not further classified, which might weaken the accuracy of results. Fourthly, due to the current limited and inconsistent data, subgroup analysis concerning some outcomes (maximal cystometric capacity, urgency) and analysis to evaluate the effect of IVES combination therapy could not be performed. Fifthly, studies concerning IVES monotherapy were of small quantity, which left the conclusions of IVES monotherapy limited. Lastly, due to the limitation of the quantity of the studies involving both PFDs and IVES, some subtypes of PFDs, like POP and sexual dysfunction, cannot be fully explored in this article. As previous systematic review have pointed out that there was insufficient evidence for or against the use of intravaginal ES therapy for women with PFD symptoms like SUI, there's a lot of space remained to be explored in these results (17). Based on the current research status in this field, follow-up research can be carried out for some specific research questions, such as exploring the setting of IVES stimulation parameters, exploring the intervention of different disease subtypes in the disease spectrum of PFDs and exploring the mechanisms behind these physiotherapies, etc. Large-scale clinical trials and network meta-analyses are needed in these fields. These limitations mentioned above may weaken the significance of this study.

## 5 Conclusion

The conclusions should be drawn carefully because of the limited evidence quality and quantity. On the one hand, current evidences tended to support the cure effect of IVES monotherapy, while heterogeneity existed in some of the outcomes. On the other hand, there wasn't sufficient evidence for or against the use of intravaginal ES therapy combined with other therapies in women with SUI, partly due to the variability in the choice of other therapies to combine with IVES. Hence, there is a need for further high-quality randomized controlled studies on the effectiveness of intravaginal ES for UI.

## Data availability statement

The original contributions presented in the study are included in the article/Supplementary material, further inquiries can be directed to the corresponding authors.

## Author contributions

RC: Conceptualization, Data curation, Investigation, Methodology, Software, Writing – original draft. RW: Data curation, Investigation, Methodology, Writing – original draft. YY: Data curation, Methodology, Writing – original draft. KZ: Conceptualization, Data curation, Formal analysis, Funding acquisition, Investigation, Methodology, Project administration, Resources, Software, Supervision, Validation, Visualization, Writing – original draft, Writing – review & editing. JL: Funding acquisition, Project administration, Supervision, Writing – review & editing.
