# Supplementary material for: Intravaginal electrical stimulation for the treatment of pelvic floor dysfunction: a systematic review and meta-analysis
Source: Front Neurol. 2024 Aug 13;15:1378494. doi: 10.3389/fneur.2024.1378494 (PMC11348806; doi:10.3389/fneur.2024.1378494)
Supplement: Supplementary file 1 [file Table_1.docx]

**Table S1. Search strategy**

| **Database** | **#** | **Search strategy** | **Results** |
| --- | --- | --- | --- |
| PubMed | 1 | "Electric Stimulation"[MeSH Terms] OR "Electric Stimulation Therapy"[MeSH Terms] | 218,763 |
|  | 2 | ("direct"[Title/Abstract] OR "puls*"[Title/Abstract] OR "high frequency"[Title/Abstract] OR "low frequency"[Title/Abstract] OR "electric*"[Title/Abstract]) AND ("current*"[Title/Abstract] OR "stimula*"[Title/Abstract]) | 350,940 |
|  | 3 | ("monophasic*"[Title/Abstract] OR "biphasic*"[Title/Abstract]) AND ("pulse"[Title/Abstract] OR "current*"[Title/Abstract]) | 6,137 |
|  | 4 | #1 OR #2 OR #3 | 500,390 |
|  | 5 | vagina[MeSH Terms] | 38,449 |
|  | 6 | intravaginal[Title/Abstract] | 5,702 |
|  | 7 | #5 OR #6 | 42,900 |
|  | 8 | (randomized controlled trial[Publication Type] OR controlled clinical trial[Publication Type] OR randomized[Title/Abstract] OR placebo[Title/Abstract] OR clinical trials as topic[Mesh:NoExp] OR randomly[Title/Abstract] OR trial[Title]) NOT (animals [Mesh] NOT (humans[Mesh] AND animals[Mesh])) | 1,400,574 |
|  | 9 | #4 AND #7 AND #8 | 79 |
|  | 1 | MeSH descriptor: [Electric Stimulation] explode all trees | 2,265 |
| Cochrane library | 2 | MeSH descriptor: [Electric Stimulation Therapy] explode all trees | 9,313 |
|  | 3 | (direct OR puls* OR "high frequency" OR "low frequency" OR electric*):ti,ab,kw AND (current* OR stimula*):ti,ab,kw | 35,379 |
|  | 4 | (monophasic* OR biphasic*):ti,ab,kw AND (pulse OR current*):ti,ab,kw | 545 |
|  | 5 | #1 OR #2 OR #3 OR #4 | 39,331 |
|  | 6 | MeSH descriptor: [Vagina] explode all trees | 1,572 |
|  | 7 | (intravaginal):ti,ab,kw | 3,465 |
|  | 8 | #6 OR #7 | 4,443 |
|  | 9 | #5 AND #8 | 125 |
| EMBASE | 1 | 'electrostimulation'/exp OR 'electrotherapy'/exp | 381,877 |
|  | 2 | (direct:ab,ti OR puls*:ab,ti OR 'high frequency':ab,ti OR 'low frequency':ab,ti OR 'electric*':ab,ti) AND (current*:ab,ti OR stimula*:ab,ti) | 438,399 |
|  | 3 | (monophasic*:ab,ti OR 'biphasic*':ab,ti) AND (pulse:ab,ti OR current*:ab,ti) | 8,115 |
|  | 4 | #1 OR #2 OR #3 | 717,585 |
|  | 5 | 'vagina'/exp | 52.326 |
|  | 6 | intravaginal:ab,ti | 7,570 |
|  | 7 | #5 OR #6 | 56,653 |
|  | 8 | 'crossover procedure':de OR 'double-blind procedure':de OR 'randomized controlled trial':de OR 'single-blind procedure':de OR random*:de,ab,ti OR factorial*:de,ab,ti OR crossover*:de,ab,ti OR ((cross NEXT/1 over*):de,ab,ti) OR placebo*:de,ab,ti OR ((doubl* NEAR/1 blind*):de,ab,ti) OR ((singl* NEAR/1 blind*):de,ab,ti) OR assign*:de,ab,ti OR allocat*:de,ab,ti OR volunteer*:de,ab,ti | 3,091,388 |
|  | 9 | #4 AND #7 AND #8 | 189 |

**Search date:** 2023/3/6
