# Supplementary figures and images for: Intravaginal electrical stimulation for the treatment of pelvic floor dysfunction: a systematic review and meta-analysis
Source: Front Neurol. 2024 Aug 13;15:1378494. doi: 10.3389/fneur.2024.1378494 (PMC11348806; doi:10.3389/fneur.2024.1378494)

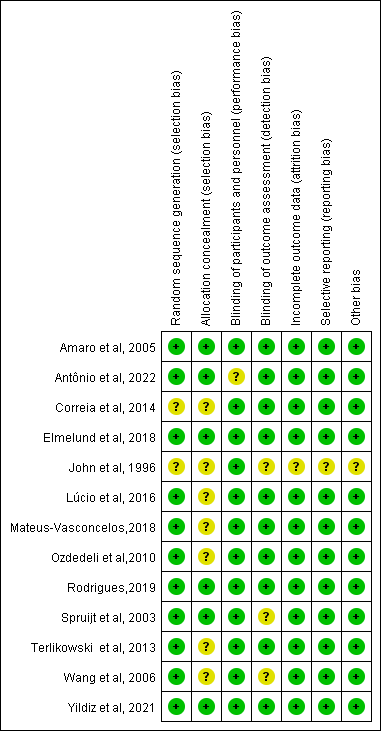

Supplement: Supplementary file 2 [file Image_1.PNG]
